# Nano Astaxanthin ameliorates myocardial infarction in rats through autophagy

**DOI:** 10.1038/s41598-025-06206-3

**Published:** 2025-06-20

**Authors:** Aliaa M. Radwan, Samar Gaber Shaybob, Ehab Tousson, Maher A Kamel, Tarek M. Mohamed

**Affiliations:** 1 Biochemistry Division, Chemistry Department, Faculty of Science, Tanta University, Tanta, 31527 Egypt; 2 Zoology Department, Faculty of Science, Tanta University, Tanta, 31527 Egypt; 3 Biochemistry Department, Medical Research Institute, Alexandria University, Alexandria, Egypt; 4 Research Projects Unit, Pharos University in Alexandria, Alexandria, 21648 Egypt

**Keywords:** Isoprenaline, Myocardial infarction, Autophagy, Astaxanthin, Nano-astaxanthin, Biochemistry, Drug discovery, Cardiology

## Abstract

Acute myocardial infarction (MI), a serious manifestation of ischemic heart disease, remains the culprit for mortality among coronary heart disease patients. Astaxanthin has demonstrated the ability to alleviate inflammation-induced myocardial damage while maintaining a balance between oxidants and antioxidants. This study investigates the cardioprotective potential of astaxanthin (ASX), particularly when encapsulated in nanostructured lipid carriers (NLCs), in isoprenaline (ISO)-induced myocardial infarction in rats. The study involved 48 rats separated into 6 groups. ASX and Nano-ASX (5 mg/kg) were administrated orally for 21 days before MI induction (isoprenaline, 85 mg/kg, subcutaneously). Blood and cardiac tissue samples were taken 24 h following the last isoprenaline injection for biochemical and histopathological investigation. The findings reveal that nano-formulated ASX significantly reduces oxidative stress and cardiac injury markers, including CK-MB, Troponin-I, and LDH. Additionally, it enhances antioxidant enzyme activities (GSH, GPx, and GSH-RD) and decreases inflammatory markers (COX-2 and VEGF). The study further demonstrates that nano-ASX stimulates autophagy by upregulating critical genes such as Beclin-1, ULK1, and LC3B, which are vital for cardiac protection and repair. Histological analysis confirms these biochemical outcomes, showing reduced myocardial damage and inflammation in the nano-ASX-treated groups. This study concludes the potential of ASX nano-formulations as an advanced therapeutic approach for myocardial infarction, leveraging improved bioavailability and targeting oxidative stress, inflammation, and autophagic mechanisms.

**Supplementary Information:**

The online version contains supplementary material available at 10.1038/s41598-025-06206-3.

## Introduction

Acute myocardial infarction (MI), a severe form of ischemic heart disease, maintains the leading global cause of death among coronary heart disease patients^1^. MI occurs due to a disparity between the coronary blood supply and the myocardial muscles’ oxygen demand, often leading to myocardial fibrosis and cardiac hypertrophy^2^. The condition is accompanying with numerous pathological changes in the myocardium, including free radical damage, thrombosis, and hyperlipidemia^3^. Enhanced formation of reactive oxygen species (ROS) in ischemic tissue is extensively reported to cause oxidative damage to lipids, proteins, carbohydrates, and DNA^4^. This oxidative stress damages cell membranes, promotes lipid peroxidation, and reduces antioxidant defenses^5^.

Isoprenaline, a β-adrenergic agonist, is known to induce MI at high doses by auto-oxidizing to generate cytotoxic free radicals. These free radicals trigger the oxidation of membrane phospholipids, producing severe damage to the cardiac membrane. Consequently, isoprenaline is frequently used in experimental models to induce myocardial infarction in rats for studying potential protective therapies^6^.

Astaxanthin, a non-vitamin A pro-carotenoid, has garnered significant scientific interest due to its potent antioxidant properties, which are ten times stronger than β-carotene and 100 times more efficient than vitamin E^7^. Numerous studies have highlighted the use of astaxanthin in providing cardioprotection against isoproterenol (ISO)-induced MI in rats, identifying mitochondrial biogenesis as a potential molecular target^8^. Another study demonstrated that astaxanthin could mitigate inflammation-induced myocardial injury while maintaining a balance between oxidants and antioxidants^9^. Similarly, Hussein found that astaxanthin offers substantial cardio protection by enhancing antioxidant enzyme activities and reducing MDA and hydrogen peroxide levels^10^. ASX poses some limitations in its therapeutic applications due to its water insolubility, low bioavailability, instability, and decomposition by oxygen and light^11^. Nanotechnology approaches overcome these limitations and enhance the bioavailability, pharmacokinetics, and pharmacodynamics of the drug^12^. As seen in most carrier-nano formulations, using some chemicals could induce side effects, and time consuming are the major challenges that face ASX- loaded on nanocarrier for drug delivery^13^. Therefore, using biodegradable carriers such as lipid carriers could solve these drawbacks.

Autophagy, a cellular energy-dependent degradation and recycling process, maintains homeostasis by breaking down cytosolic organelles and long-lived proteins^14^. In the heart, basal autophagy preserves cardiac structure and function, playing a critical role during acute and chronic ischemia, where it supports recovery following normalized coronary flow. In ischemic regions, autophagy acts as a protective mechanism, aiding in the survival of hibernating myocardium and shielding cardiomyocytes from ischemic death^15^. Accordingly, this study aimed to appraise the cardioprotective effects of astaxanthin encapsulated in solid lipid nanoparticles (SLNs) against isoprenaline-induced myocardial infarction in rats and investigate autophagy as a potential molecular mechanism of its therapeutic action.

## Materials and Methods

### Chemicals and drugs

Isoprenaline HCl (Cat No. FI33495) and Astaxanthin (95%, Cat No. FA18001) were purchased from Biosynth company.

### Preparation and characterization of Astaxanthin (ASX)-loaded nanostructured lipid carriers (nano-ASX)

#### Preparation of nano-ASX

ASX-loaded nanostructured lipid carriers (NLCs) were prepared using the hot high-pressure homogenization (HPH) technique with glyceryl monostearate (GMS) as the solid lipid, oleic acid as the liquid lipid, and Tween 80 as the surfactant^16^. GMS was melted at 50 °C, followed by the addition of oleic acid to the molten lipid while maintaining the temperature. The lipid mixture contained 1.5% (w/v) total lipids with a solid-to-liquid lipid ratio of 90:10. Next, 40 mg of ASX was added to the molten lipid phase. The aqueous phase, consisting of 1.5% (w/v) Tween 80 dissolved in distilled water, was added to the molten lipid under continuous magnetic stirring at 1000 rpm for 20 min. This mixture was then homogenized at high speed (20,000 rpm) for 15 min using a digital ultra-TURRAX (IKA^®^ T25, Germany). The resulting nano-emulsion was cooled overnight at 4–8 °C to allow recrystallization and NLC formation.

### Characterization of prepared drug-loaded NLCs

The particle size (PS), polydispersity index (PDI), and zeta potential (ZP) of the ASX-loaded NLCs were measured by a Malvern Zetasizer (Malvern Instruments, UK). The readings were taken in triplicate after diluting the samples with distilled water, and the results were presented as mean ± standard deviation (SD)^17^.

### Stability study of ASX-loaded NLCs

The stability of the ASX-loaded NLCs was assessed over three months at storage temperatures of 4 °C and 25 °C. Particle size measurements were compared to baseline (freshly prepared NLCs) to evaluate stability.

### Entrapment efficiency (%EE) of ASX-loaded NLCs

Entrapment efficiency (%EE) of ASX-loaded NLCs was determined using centrifugal ultrafiltration. A 5 mL sample of the ASX-Nano formulation was centrifuged at 10,000 rpm for 20 min at 4 °C. The supernatant was filtered and analyzed for free, unencapsulated ASX using high-performance liquid chromatography (HPLC; Agilent Technologies 1200 Infinity Series, USA). The %EE was calculated using the formula:

%EE = [(Total ASX in 5 mL NLC) - (Free ASX in supernatant)]/(Total ASX in 5 mL NLC).

### In vitro drug release and release kinetic studies

In vitro release study was carried out to evaluate the release of ASX from the optimized ASX – NLCs formulation and comparing it with the pure drug. It was performed by dialysis bag diffusion technique employing a dialysis membrane (supporting information for details). Comparison between release profile of ASX solution and ASX-NLCs was made by simple Student’s t test at *p* < 0.05. In vitro release data of ASX-NLCs formulation was fitted to various kinetic equations; zero order, first order, Higuchi diffusion, Hixon-Crowell, and Korsmeyer - Peppas models. The correlation coefficients (r^2^) were computed to indicate applicability of the model to the release data.

## In vivo study

### Animals

Forty-eight healthy adult male albino rats (150–200 g) were taken from the Medical Research Institute, Alexandria University, Egypt. The rats were housed under a 12:12 light/dark cycle with free access to food and water. All experimental procedures were conducted in compliance with the NIH Guidelines for the Care and Use of Laboratory Animals (NIH Publications No. 8023, revised 1978) and were approved by the IACUC of Tanta University (Approval No. IACUC-SCI-TU-0286). The study adhered to ARRIVE guidelines and the National Research Council’s animal care standards.

### Induction of myocardial infarction (MI)

Myocardial infarction was produced by subcutaneous injection of isoprenaline (ISO) at 85 mg/kg, dispersed in physiological saline, administered in the right thigh of each rat for two successive days with a 24-hour interval^18^.

### Experimental design

The rats were separated into two main groups: **Control Group** (16 rats): Subdivided into control rats and rats supplemented with ASX solution (5 mg/kg) daily via oral gavage for 21 days^8,19^. **MI Group** (32 rats): Subdivided into untreated MI rats, MI rats pretreated with the nanocarrier vehicle (MI + Vehicle), MI rats pretreated with ASX solution (MI + ASX), and MI rats pretreated with nano ASX (MI + nano ASX). The treatments (5 mg/kg via oral gavage) were administered daily for 21 days before MI induction.

### Sample preparation

At the experiment’s conclusion, rats were fasted for 12 h, weighed, and anesthetized using isoflurane (1–4%). They were then sacrificed by cervical dislocation, and blood was collected for serum analysis. Hearts were excised, rinsed with saline, and homogenized in phosphate-buffered saline (PBS, pH 7.4) at a 1:10 ratio. Homogenates were centrifuged (10,000 ×g, 4 °C, 20 min), and supernatants were stored at −20 °C for further analysis. Tissue samples (30 mg) were used for RNA extraction.

### Biochemical measurements

Serum levels of Troponin-I were measured using ELISA kits (CSB-E08594r, Cusabio, USA), while ALT, AST, LDH, creatine kinase (CK-MB), urea, and creatinine were analyzed using commercial kits (Spectrum, Egypt). Cardiac glutathione level was measured using previously reported method^20^. The lipid peroxidation level in cardiac homogenate was assessed by evaluating malondialdehyde (MDA) production as a marker of oxidative stress^21^. Furthermore, myocardial nitric oxide (NO) was detected using a colorimetric assay kit (Cat No. E-BC-K035-M, Elabscience, USA) following manual methods. The activity of glutathione reductase (GSH-RD) and glutathione peroxidase (GPx) were detected using commercial ELISA kits (Cat No. ABIN6963266, MBS1600722). In addition, VEGF-A and COX-2 levels were quantified using specific ELISA kits (Cat No. MBS266603, E-EL-R2603, Elabscience, USA) as described in the detection kit’s user manual.

### Gene expression assessment

 Total mRNA was isolated from heart tissues using the RNeasy Mini Kit (Qiagen, Germany). Reverse transcription was carried out with the TOPscript™ RT DryMIX kit (Enzynomics Co Ltd, Korea). Expression levels of autophagic genes (Beclin1, LC3B, ULK1) were quantified using RT-qPCR with gene-specific primers (Table 1). Data normalization and fold-change analysis were conducted using the 2^−ΔΔCt^ method^22^.

**Table 1:** Primers sequences used in this study.

Gene	Accession No.	Primer sequence	
GAPDH	NM_017008.4	F	GGGTGTGAACCACGAGAAATA
		R	AGTTGTCATGGATGACCTTGG
Beclin1	NM_001034117.1	F	TTGGCCAATAAGATGGGTCTGAA
		R	TGTCAGGGACTCCAGATACGAGTG
LC3B	NM_022867.2	F	CAGGATCCATGCCGTCCCAGAAGACC
		R	GTCCCTTTTTGCCTTGGTAG
ULK1	NM_001108341.1	F	CATCCGAAGGTCAGGTAGCA
		R	GATGGTTCCCACTTGGGGAGA

### Histopathological assessment

 After necropsy, cardiac tissue samples from various rat groups were collected, rinsed in saline, and fixed in 10% neutral buffered formalin (pH 7.4) for a minimum of 24 h. The fixed tissues were processed using the paraffin embedding method. Thin Sect. (4.5 μm) were prepared, dewaxed, and stained with Mayer’s hematoxylin and eosin (H&E). The sections were then dehydrated using alcohol, cleared with xylene, and sealed. The stained tissue slides were examined under a light microscope (Leica DM500) and photographed using a digital camera (Leica EC3, Germany**)**^23^.

### Statistical analysis

The data were analyzed using the GraphPad prism software package (version 8.0.1, 244). Results were expressed as mean ± SD, and comparisons between groups were performed using ANOVA followed by post-hoc Tukey’s tests. Statistical significance was determined at a *P* value threshold of < 0.05.

## Results

### Characterization of ASX-loaded NLC

The results of PS, PDI and ZP are presented in (Table 2). It was observed that the PS of the prepared ASX-loaded NLC was about 142.9 nm and is suitable for cardiovascular targeting. PDI value of the prepared formulations was found to be less than 0.3 indicating high uniformity in particle size distribution. The prepared drug-loaded NLCs were found to be of high ZP value indicating good stability.

**Table 2:** Particle size (PS), polydispersity index (PDI), and zeta potential (ZP) of ASX– NLCs.

NLCs	PS (nm)	PDI	ZP (mV)
ASX – NLCs	142.9 ± 4.35	0.224 ± 0.07	**–** 30.3 ± 4.67

### Entrapment efficiency (% EE) and stability study of ASX-loaded NLC

The ASX drug showed very high entrapment efficiency (EE%); about 92.4 ± 3.57%, in the obtained NLC-formulation. In addition, the stability study of ASX-loaded NLC was shown in Table 3**.** Slight change was observed in particle size of the prepared ASX-loaded NLC when they were stored at 4^◦^C (refrigerator) up to three months, while higher increase in particle size was detected when they were stored at 25^◦^C (room temperature). This could be attributed to an increase in the kinetic energy of the system as a result of high temperature when the formulations were stored at room temperature, which could accelerate collision of particles leading to particle aggregation during storage. Therefore, storage of ASX-loaded NLC is preferred to be at 4^◦^C (refrigerator).

**Table 3:** Particle size (PS) of the stored ASX-NLCs under different storage conditions (at refrigerator 4◦C and room temperature 25◦C) after 3 months of storage.

		PS (nm)
		ASX – NLCs
At Zero time		142.9 ± 4.35
After 3 months	at 4 ◦C	151.2 ± 5.35
	at 25 ◦C	174.7 ± 6.94

### In vitro release and release kinetic studies

**Fig. 1:**
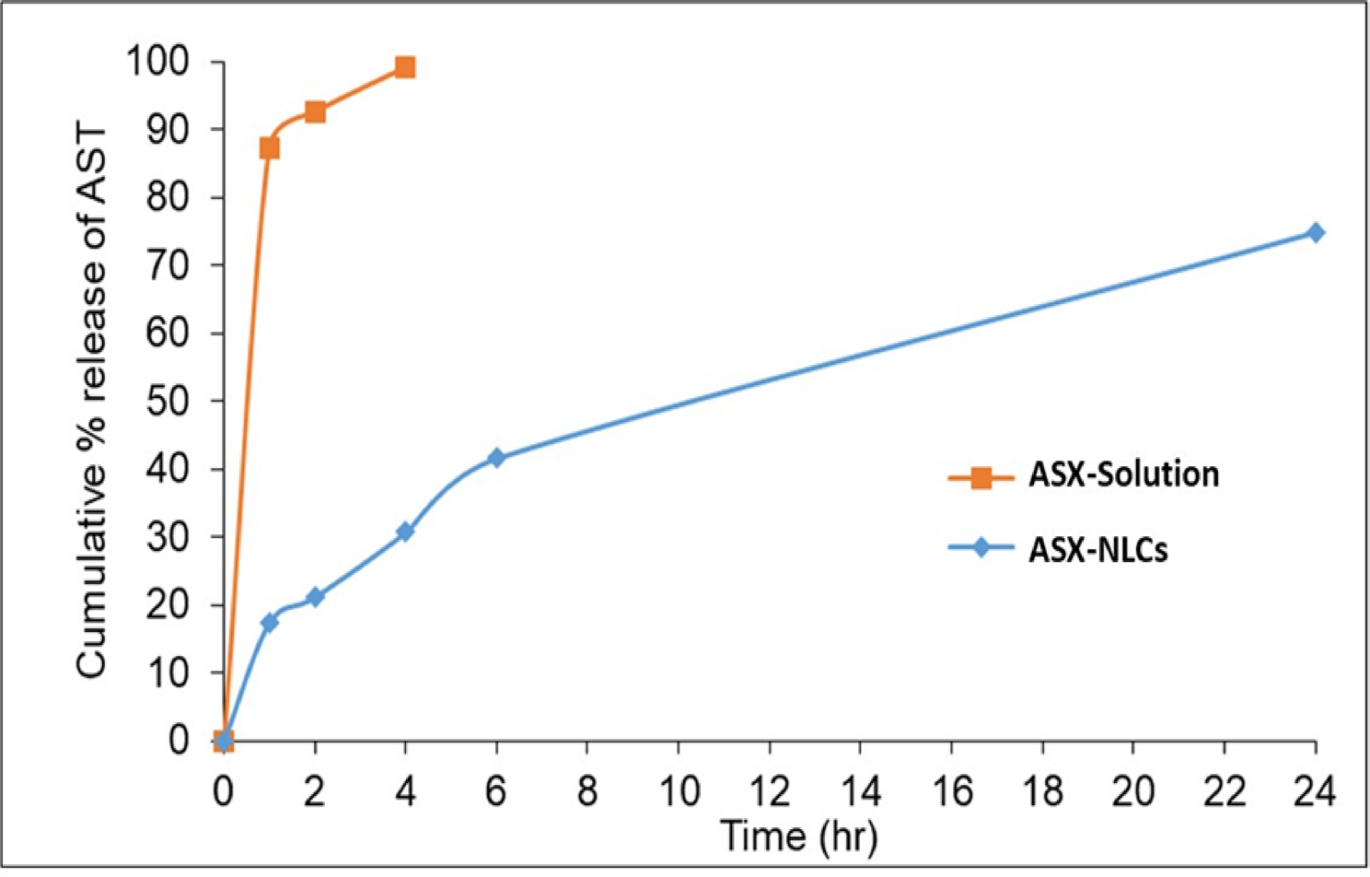
In vitro release profile of Astaxanthin from ASX – Solution and ASX–NLCs for 24 using dialysis method in phosphate buffer pH 7.4 at 37 ± 0.5 °C.

 As seen from Fig. 1, the release of AS from ASX–NLCs formulation demonstrated a biphasic release with an initial rapid release (about 17% of ASX released in the first hour), followed by slow and sustained release in the second phase of 75% up to 24 h. On the other hand, ASX-Solution showed immediate release of 87.3% of the drug only in the first hour and almost all the drug content 99.1% released in 4 h.

The biphasic release behavior of NLCs; the initial burst release may be attributed to the presence of free drug in the external phase and adsorbed drug onto the surface of particles or could be attributed to the drug present in the liquid lipid, while the slow release may be owed to the encapsulated drug within the solid lipid matrix. Drug diffusion through the liquid lipid phase was more rapid than that along the solid the lipid phase. On comparing the observed results, a significance difference (*p* < 0.05) was marked between ASX–Solution and ASX–NLCs formulation after 4 h. Korsmeyer-Peppas model was found to be the best fitted model for ASX-NLCs formulation with the highest value of correlation coefficient (r^2^ = 0.9959) (Table 4). The value of release exponent “n” was found to be 0.476, which appears to indicate a non-Fickian diffusion and combination mechanism of diffusion and erosion.

**Table 4:** In vitro release kinetics parameters of ASX–NLCs formulation.

	Zero order	First order	Higuchi	Hixon-Crowell	Korsmeyer-Peppas
(K) Release rate constant	**K0**	**K1**	**KH**	**KHC**	**KHP**
	3.523	0.082	15.611	0.022	16.587
(r^2^) correlation coefficient	0.6402	0.9188	0.9945	0.8483	**0.9959**
n: release exponent in Korsmeyer-Peppas model					**0.476**

### Effect of ASX and nano-ASX on serum biochemical parameters in isoprenaline induced myocardial infarction in rats

The biochemical parameters are summarized in Table 5. Isoprenaline-induced myocardial infarction (MI) in rats resulted in a significant increase (*P* < 0.0001) in ALT and AST activity, as well as elevated urea levels, compared to the control group. Treatment with ASX or nano-ASX restored ALT activity and urea levels to near-normal values. AST activity was also significantly reduced (*P* < 0.0001) in MI rats pretreated with ASX or nano-ASX compared to untreated MI rats. However, there were no significant changes in serum creatinine levels in MI rats administered ASX or nano-ASX. Additionally, isoprenaline administration significantly elevated LDH and CK-MB activity, as well as troponin-I levels, by 184.8%, 209.4%, and 2162.8%, respectively, compared to the control group. Pretreatment with ASX or nano-ASX reduced LDH activity by 20.4% and 51.8%, respectively, and suppressed CK-MB activity by 34.2% and 60.4%, respectively, compared to untreated MI rats. Furthermore, serum troponin-I levels were reduced by 65.8% and 78.2% following ASX and nano-ASX treatment, respectively. No significant changes were observed in any of these parameters in the vehicle-treated MI rats.

**Table 5:** Effect of astaxanthin (ASX) and nano astaxanthin (Nano ASX) on liver, kidney, and cardiac functions in isoprenaline-induced myocardial infarction in rats.

Parameters	Control	ASX	MI	MI + Vehicle	MI + ASX	MI + Nano-ASX
ALT (U/L)	46.8 ± 7.49	41.2 ± 3.83	85.8^ab^ ± 14.18	70.25^ab^ ± 20.17	47.2^cd^ ± 7.19	46.5^cd^ ± 3.41
AST (U/L)	109.8 ± 13.9	109 ± 9.11	233.2^ab^ ± 20.2	226^ab^ ± 9.89	218.6^ab^ ± 18.6	190^abcd^ ± 16.5
Urea (mg/ml)	38.8 ± 3.6	35 ± 4	59^ab^ ± 4	56^ab^ ± 11	48.4^b^ ± 7.4	32.5^cde^ ± 5.3
Creatinine (mg/ml)	0.696 ± 0.08	0.736 ± 0.34	0.736 ± 0.04	0.84 ± 0.1	0.686 ± 0.056	0.715 ± 0.03
LDH (U/L)	309.6 ± 53.5	324.6 ± 51.3	881.8^ab^ ± 140.9	929.75^ab^ ± 117	701.2^abd^ ± 93	424.25^cde^ ± 76
CK-MB (U/L)	563.6 ± 106.4	497.2 ± 74.6	1744^ab^ ± 142	1908^ab^ ± 49	1146^abcd^ ± 174	690^cde^ ± 120
Troponin-I (ng/ml)	0.097 ± 0.026	0.12 ± 0.02	2.195^ab^ ± 0.120	2.4775^abc^ ± 0.186	0.75^abcd^ ± 0.11	0.477^abcd^ ± 0.083

Data presented as Mean ± SD (N = 6). ^a^significantly differs compared with control rats, ^b^significantly differs compared with ASX rats, ^c^significantly differs compared with MI, ^d^significantly differs compared with MI + Vehicle.

### Effect of ASX and nano-ASX on myocardial lipid peroxidation and antioxidant parameters

**Fig. 2:**
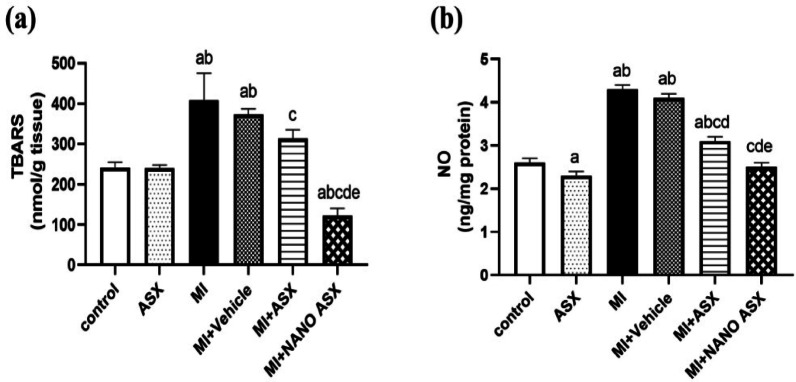
Effect of astaxanthin (ASX) and nano astaxanthin (NANO ASX) on TBARS (a) and NO (b) in cardiac tissue after isoprenaline administration. Results are expressed as Mean ± SD (N = 6). p < 0.05 is considered significant, where ^a^ significantly different compared with normal control group, ^b^ significantly different compared with ASX group, ^c^ significantly different compared with MI control group, ^d^ significantly different compared with MI + Vehicle group, and ^e^ significantly different compared with MI + ASX group. TBARS: Thiobarbituric acid-reactive substance, NOx: nitric oxide.

**Fig. 3:**
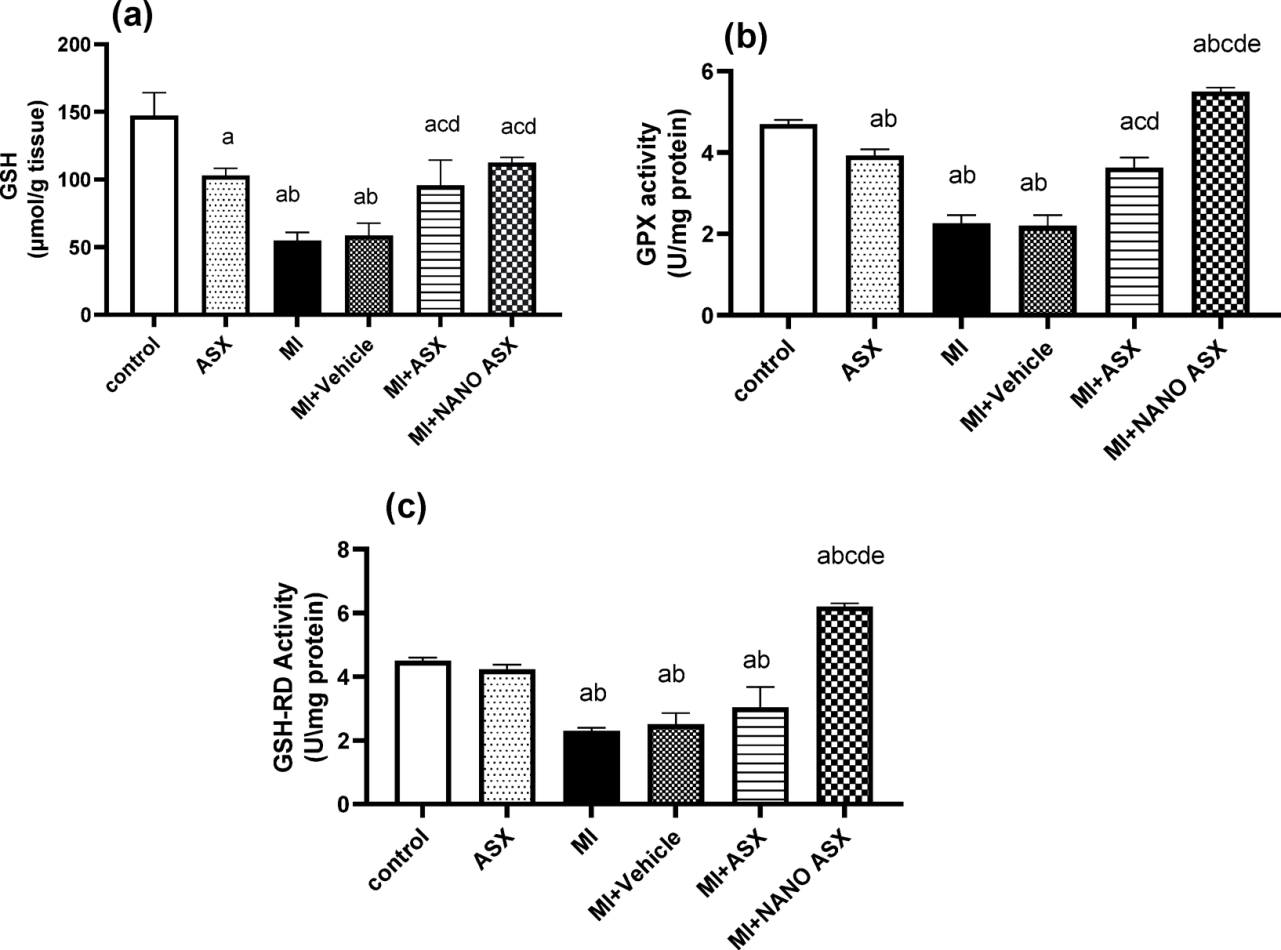
Effect of astaxanthin (ASX) and nano astaxanthin (NANO ASX) on GSH (a), GPx activity (b), and GSH-RD activity (c) in cardiac tissue after isoprenaline administration. Results are expressed as Mean ± SD (N = 6). p < 0.05 is considered significant, where ^a^ significantly different compared with normal control group, ^b^ significantly different compared with ASX group,^c^ significantly different compared with MI control group, ^d^ significantly different compared with MI + Vehicle group, and ^e^ significantly different compared with MI + ASX group, GSH: reduced glutathione, GPx: glutathione peroxidase, GSH-RD: glutathione reductase.

 The effects on lipid peroxidation and antioxidant parameters are illustrated in Figs. 2 and 3. Isoprenaline administration (MI group) caused a substantial increase (*P* < 0.0001) in cardiac TBARS and NO levels compared to the control group. Pretreatment with ASX or nano-ASX reduced TBARS levels by 23.2% (*P* = 0.0104) and 70.07% (*P* = 0.0006), and NO levels by 27.9% and 41.86%, respectively.

In addition, isoprenaline significantly decreased cardiac GSH content and the activities of GPx and GSH-RD by 62.6%, 51.4%, and 48.8%, respectively, as compared to the control group. Treatment with ASX or nano-ASX markedly increased GSH content by 73.7% and 103.9%, respectively, and significantly enhanced GPx and GSH-RD activity (*P* < 0.0001) compared to untreated MI rats. These findings highlight the cardioprotective potential of ASX, particularly in its nano-formulated form, mitigating oxidative stress and restoring antioxidant protective systems in isoprenaline-induced myocardial infarction.

### Effect of ASX and nano ASX on VEGF and COX-2 activity in isoprenaline-induced myocardial infarction in rats

**Fig. 4:**
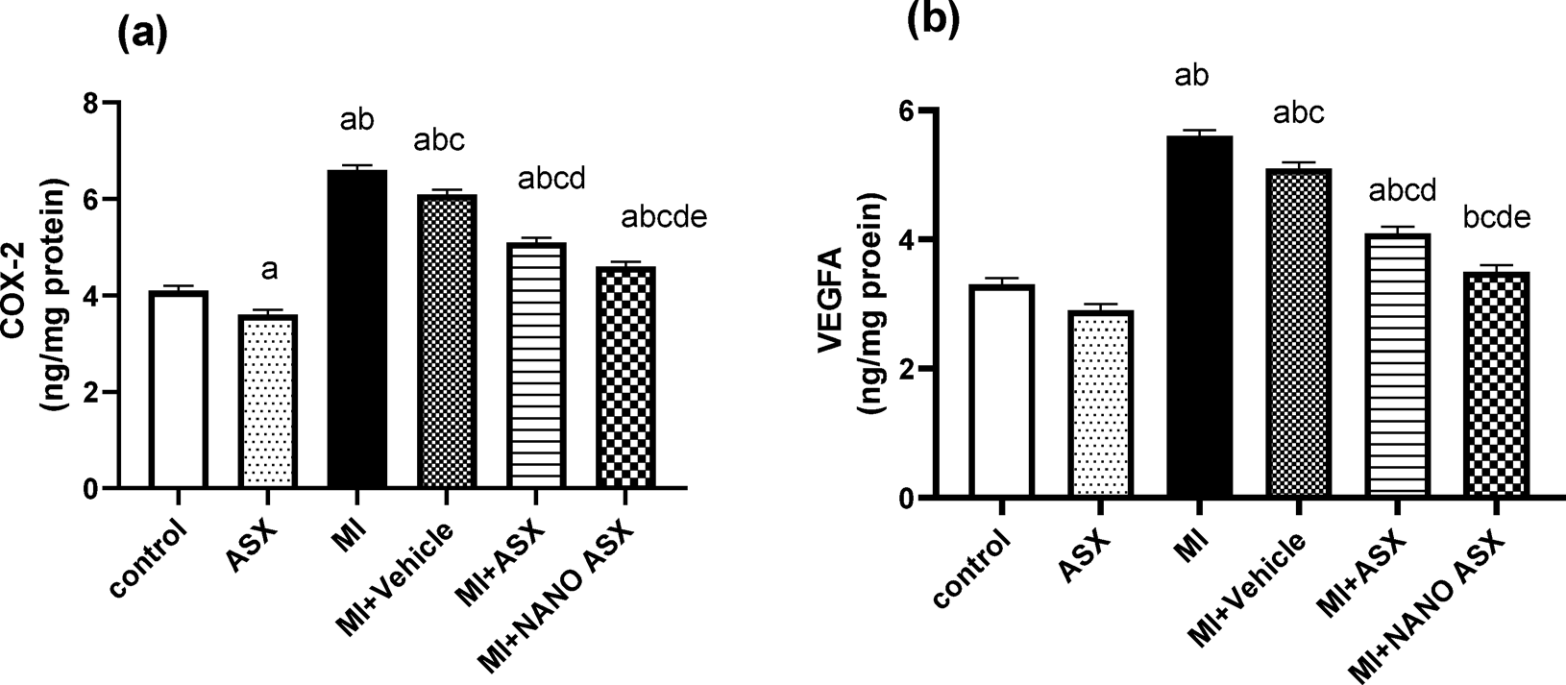
Effect of astaxanthin (ASX) and nano astaxanthin (NANO ASX) on COX-2 (a) and VEGFA (b) in cardiac tissue after isoprenaline administration. Results are expressed as Mean ± SD (N = 6). p < 0.05 is considered significant, where ^a^ significantly different compared with normal control group,^b^ significantly different compared with ASX group, ^c^ significantly different compared with MI control group, ^d^ significantly different compared with MI + Vehicle group, and ^e^ significantly different compared with MI + ASX group. COX-2: cyclooxygenase 2, VEGFA: vascular endothelial growth factor A.

 The findings revealed that isoprenaline administration considerably (*P* < 0.0001) elevated the activity of COX-2 and VEGF in comparison with the control group. The activities of these markers decreased by 22.7 and 26.7% in rats pretreated with ASX and by 30.3% and 37.5% respectively in rats pretreated with nano ASX compared to MI group (Figure 4).

### Effect of ASX and nano ASX on Beclin-1, ULK1 and LC3B mRNA expression in isoprenaline-induced myocardial infarction in rats

**Fig. 5:**
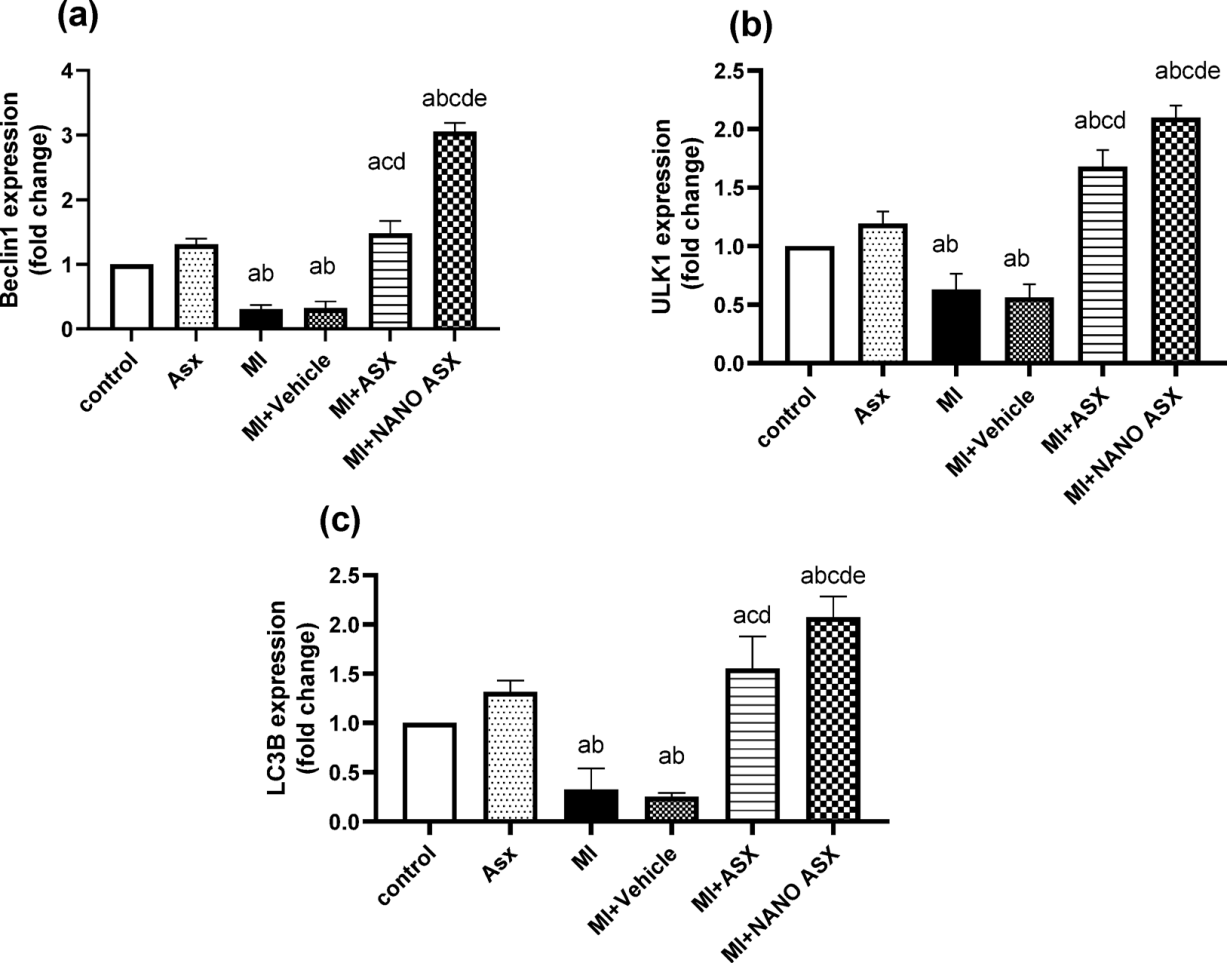
Effect of astaxanthin and nano astaxanthin on the regulation of autophagy. The mRNA levels of Beclin1 (a), ULK1 (b), and LC3B (c) were measured in cardiac tissue after isoprenaline administration using RT-PCR. Results are expressed as Mean ± SD (*N* = 6). *p* < 0.05 is considered significant, where ^a^ significantly different compared with normal control group, ^b^ significantly different compared with ASX group, ^c^ significantly different compared with MI control group, ^d^ significantly different compared with MI + Vehicle group, and ^e^ significantly different compared with MI + ASX group.

The data in Figure 5 revealed that injection of isoprenaline significantly (*P* < 0.002) downregulated Beclin-1, ULK1 and LC3B mRNA expression compared with the control and ASX groups. However, pretreatment of rats with ASX or nano ASX markedly (*P* < 0.0001) upregulated Beclin-1, ULK1 and LC3B mRNA expression compared to rats received isoprenaline. The group that was treated with nano ASX demonstrated the best results. In contrast, rats given the vehicle displayed no significant change in mRNA expression of these genes when compared to the MI group.

### Effect of ASX and nano-ASX on histopathologic alterations correlated with isoprenaline-induced myocardial infarction in rats

**Fig. 6:**
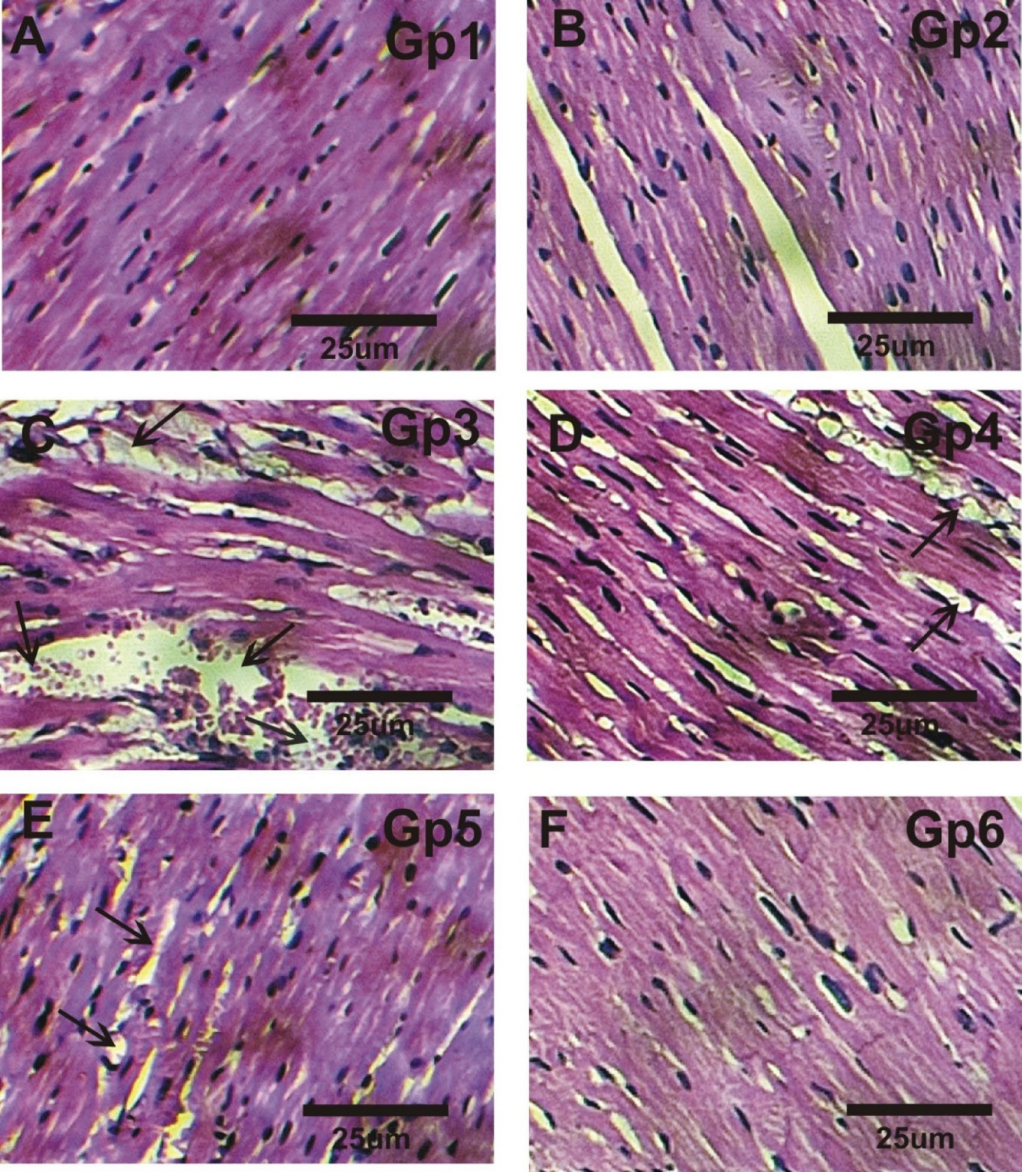
Effect of astaxanthin and nano astaxanthin on cardiac histopathologic alterations in isoprenaline-induced myocardial infarction in rats. **(A**,** B)** Heart sections of the control and ASX Gps exhibit normal myofibrillar morphology and the connective tissue, holding and supporting myocytes, contains fibroblasts. **(C)** Isoprenaline treated rat (MI Gp) revealed marked myocardial degeneration with marked inflammatory infiltration, and severe interstitial edema. **(D)** vehicle-treated rat (MI + Vehicle) revealed marked degree of myocardial injury with marked myocardial hypertrophy and moderate inflammatory infiltration. **(E)** Astaxanthin -treated rat (MI + ASX) showed a decrease in focal areas of edema and inflammatory cells infiltration. **(F)** Nano astaxanthin-treated rat (MI + nano ASX Gp) revealed mild degree of myocardial injury with only mild to moderate myocardial hypertrophy.

 Heart sections from control and ASX-treated groups showed normal myofibrillar morphology with clear striations and continuity (Fig. 6 A & B). In contrast, MI rats exhibited severe myocardial damage, including myocardial hypertrophy, fibrosis, inflammatory infiltration, interstitial edema, nuclear pyknosis, cytoplasmic vacuolization, and disrupted striations (Fig. 6 C). Vehicle-treated MI rats displayed severe myocardial injury and moderate inflammation (Fig. 6D). MI rats pretreated with ASX (MI + ASX) showed reduced edema and inflammatory infiltration, as well as moderate myocardial hypertrophy (Fig. 6E). Notably, mild to moderate myocardial hypertrophy and minimal myocardial damage was seen in MI rats pretreated with nano-ASX (MI + nano-ASX) (Fig. 6 F)

.

## Discussion

Isoprenaline-induced myocardial infarction (MI) in animal models is widely used due to its simplicity, speed, and minimal complications. Isoprenaline acts non-selectively on β1 and β2 receptors, with β1 receptors predominantly found in the heart, leading to positive chronotropic, dromotropic, and inotropic effects^24^. However, ischemia occurs as a result of the imbalance between heightened heart activity and reduced coronary blood flow caused by its depressor effects^25^. This study intended to evaluate the cardioprotective effects of astaxanthin (ASX) and its nano-formulated version (nano-ASX) on isoprenaline-induced myocardial infarction and to explore the molecular mechanisms of autophagy involved.

Isoprenaline-generated free radicals initiate peroxidation of membrane-bound polyunsaturated fatty acids, resulting in structural and functional heart damage along with changes in membrane permeability. Increased membrane permeability or myocyte death leads to the leakage of cytosolic contents into systemic circulation, where they serve as biomarkers of ischemic heart disease^26^. Diagnostic markers such as Troponin-I (CTn-I) and CK-MB are released during necrosis, indicating plasma membrane disruption^27^.In this study, isoprenaline administration significantly elevated cardiac enzymes CK-MB, Troponin-I, and LDH. These results align with previous studies^28^^29^;. that also reported increased levels of these markers post-MI induction. Pretreatment with ASX reduced the levels of these markers, while nano-ASX normalized them to near-control levels, indicating superior cardio protection. Isoprenaline also elevated liver (ALT, AST) and kidney (urea) biomarkers. While ASX slightly reduced these levels, nano-ASX treatment significantly lowered AST and urea, confirming its enhanced efficacy^30^.

Oxidative stress, a key contributor to myocardial infarction, manifests as an imbalance between oxidants and antioxidants^31^. ROS-mediated oxidative stress contributes significantly to ISO cardiotoxicity by interfering with the antioxidant system and affecting antioxidant enzymes, resulting in oxidative damage to cardiac tissues. The results revealed that ISO can reduce GSH level, inhibit GPx and GSH-RD activities, resulting in accumulation of MDA and NO lipid peroxidation products. Our findings are consistent with earlier studies^32,33^. In our work, ASX and nano ASX reduce the toxicity of heart induced by ISO effectively by improving the antioxidant capacity and decreasing the oxidative stress degree. These findings coincide with Mahmoud et al.‘s observation that astaxanthin alleviated isoproterenol-induced myocardial infarction by scavenging free radicals and reduced oxidative damage in cardiac tissue^8^.

Hypoxia is the primary cause of cardiomyocyte damage, and it also leads to aberrant vascular remodeling and cardiac abnormalities. ISO causes more demand for oxygen and diminishes oxygen delivery, leading to severe myocardial hypoxia. In hypoxic conditions, there is significant elevation in HIF-1α and its downstream target genes including angiogenic factor, vascular endothelial growth factor (VEGF)^34^. In this work, ISO administration considerably increases VEGF level. Our findings are comparable with those of Viswanadha et al., who found that isoproterenol stimulated hypoxia-driven angiogenesis in myocardial ischemia damage via upregulating VEGF expression^35^. ASX and nano ASX pretreatment significantly suppressed the level of VEGF in ISO-induced myocardial infarction. Furthermore, ASX pretreatment reduced COX-2 inflammatory marker levels compared to ISO-treated rats, with nano ASX achieving a considerable reduction, indicating that it has increased anti-inflammatory and anti-angiogenic capabilities^36,37^.

Autophagy is a defensive mechanism that increases cell survival and is critical for cardiac protection. The process involves key markers like Beclin-1, ULK1, and LC3B. Previous research indicated that Beclin-1 was associated with mitophagy in cardiac tissue through PINK-Parkin pathway^38^. ULK-1 protects the heart towards cardiac dysfunction and mediates mitochondrial maintenance through mitophagy^39^. In the existing endeavor, isoprenaline significantly downregulated these autophagy-related genes. Pretreatment with ASX upregulated their expression, with nano-ASX producing the most pronounced effects. Previous studies indicated that autophagy helps maintain heart function by preventing aggresome harmful impact of protein accumulation^40^. Further, deletion or down regulation of autophagy genes could associate with cardiac malfunction. Kaizuka et al., indicate that deletion of ULK1 in mice was associated with lipotoxicty with cardiac malfunction^41^. We concluded that ASX and its nano form could induce autophagy to protect against myocardial infarction via preserving cellular homeostasis and breaking down organelles or improperly folded proteins to produce ATP in cardiomyocytes, as also highlighted by previous studies^42,43^. However, conducting LC3-II/LC3-I ratio and p62 protein degradation level were recommended to elucidate that functional autophagy take place.

## Conclusion

Overall, this study underscores the potential of nano-ASX as a superior therapeutic agent for myocardial infarction, offering enhanced protection against oxidative stress, inflammation, and impaired autophagy compared to conventional ASX formulations.

## Electronic supplementary material

Below is the link to the electronic supplementary material.

Supplementary Material 1

## Data Availability

The datasets used and/or analyzed during the current study are available from the corresponding author on reasonable request.
