# Supplementary material for: Nano Astaxanthin ameliorates myocardial infarction in rats through autophagy
Source: Sci Rep. 2025 Jun 20;15:20195. doi: 10.1038/s41598-025-06206-3 (PMC12181253; doi:10.1038/s41598-025-06206-3)
Supplement: Supplementary file 1 — Supplementary Material 1 [file 41598_2025_6206_MOESM1_ESM.docx]

**Supporting information**

**Nano Astaxanthin Ameliorates Myocardial Infarction in Rats through Autophagy**

**Aliaa M. Radwan^1*^, Samar Gaber Shaybob^1^, Ehab Tousson^2^, Maher A Kamel^3,4^, Tarek M. Mohamed^1*^**

*^1^ Biochemistry Division, Chemistry Department, Faculty of Science, Tanta University, Tanta* 31527*, Egypt*

alyaa_radwan@science.tanta.edu.eg

[tarek.ali@science.tanta.edu.eg](mailto:tarek.ali@science.tanta.edu.eg)

samar152388@science.tanta.edu.eg

*^2^ Zoology Department, Faculty of Science, Tanta University, Tanta 31527, Egypt*

ehabtousson@science.tanta.edu.eg

*^3^ Biochemistry Department, Medical Research Institute, Alexandria University, Alexandria, Egypt*

*^4^ Research Projects unit, Pharos University in Alexandria; 21648, Alexandria, Egypt*

maher.kamel@alexu.edu.eg

**^*^Corresponding author**

**Aliaa M. Radwan**

[alyaa_radwan@science.tanta.edu.eg](mailto:alyaa_radwan@science.tanta.edu.eg)

**Tarek M. Mohamed**

[tarek.ali@science.tanta.edu.eg](mailto:tarek.ali@science.tanta.edu.eg)

***In vitro* drug release and release kinetic studies**

*In vitro* release study was carried out to evaluate the release of ASX from the optimized ASX – NLCs formulation and comparing it with the pure drug. It was performed by dialysis bag diffusion technique employing a dialysis membrane (Dialysis tubing Visking®: regenerated cellulose MWCO 12-14, Medicell International Ltd, London, UK). An accurate amount of ASX - NLCs and ASX solution (contain the same ASX concentration in ASX-NLCs) was transferred to dialysis bags and sealed at both ends. The sealed bags were then suspended in a beaker containing 100 mL of phosphate buffer (pH 7.4 , corresponding to physiological pH) , placed in thermostatically controlled shaking water bath (type3047, Kottermann, Hanigsen, Germany), and stirred at a constant speed (100 rpm) at 37 ± 0.5°C. Aliquots (5 mL) were withdrawn at predetermined time intervals up to 24 hr from receiver compartment (beaker) and replaced with an equal volume of fresh medium to maintain sink condition. The samples were analyzed by HPLC method to determine the amount of ASX released. Percent cumulative drug released versus time was plotted to illustrate the drug release pattern. Comparison between release profile of ASX solution and ASX-NLCs was made by simple Student’s t test at p < 0.05. In vitro release data of ASX-NLCs formulation was fitted to various kinetic equations; zero order, first order, Higuchi diffusion, Hixon-Crowell, and Korsmeyer - Peppas models. The correlation coefficients (r^2^) were computed to indicate applicability of the model to the release data. To find out the mechanism of ASX release from ASX - NLCs, in vitro release data of ASX-NLCs was fitted in the Korsmeyer - Peppas model ( Mt/M∞= Ktn ) and value of n (release exponent) was determined.

**Table (S1): ProTox 3.0 - Prediction of Astaxanthin Toxicity**

| **Name** | **Astaxanthin** |
| --- | --- |
| Molweight | 596.84 |
| Number of hydrogen bond acceptors | 4 |
| Number of hydrogen bond donors | 2 |
| Number of atoms | 44 |
| Number of bonds | 45 |
| Number of rotable bonds | 10 |
| Molecular refractivity | 187.16 |
| Topological Polar Surface Area | 74.6 |
| Octanol/water partition coefficient (logP) | 8.91 |
| Predicted LD50 | 4600mg/kg |
| Predicted toxicity class | 5 |


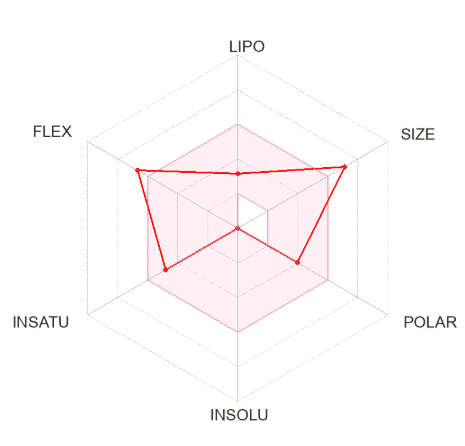


**Figure (S1):** Astaxanthin drug radar obtained Swiss ADME online server
